# Impact of Violations of the Shortest Distance-Based Transport Protocol for Intra-Arrest on Clinical Outcomes in a Metropolitan City: A Large-Scale Registry Study

**DOI:** 10.3390/jcm15031282

**Published:** 2026-02-05

**Authors:** Ju Hwan Choi, Arom Choi, Hanna Yoon, Chaeryoung Park, Soyoung Jeon, Eunju Lee, Ji Hoon Kim

**Affiliations:** 1 Department of Emergency Medicine, Gangnam Severance Hospital, Yonsei University College of Medicine, Seoul 06273, Republic of Korea; pungu73@yuhs.ac; 2 Department of Emergency Medicine, Severance Hospital, Yonsei University College of Medicine, Seoul 03722, Republic of Korea; noonup7@yuhs.ac (H.Y.); chaeryoungpark@yuhs.ac (C.P.); 3 Yonsei Institute for Digital Healthcare, Yonsei University, Seoul 03722, Republic of Korea; 4 Biostatistics Collaboration Unit, Yonsei University College of Medicine, Seoul 06273, Republic of Korea; jsy0331@yuhs.ac (S.J.); dmswn1578@yuhs.ac (E.L.)

**Keywords:** cardiac arrest, emergency medical services, resuscitation, transport protocol

## Abstract

**Background/Objective**: The optimal strategy for hospital transport of patients with out-of-hospital cardiac arrest is unclear. A transport protocol based on the shortest travel route was implemented in a metropolitan area in the Republic of Korea to minimize prehospital transport time; however, the protocol is frequently violated. This study evaluated whether protocol violations influenced the clinical outcomes of patients who experienced intra-arrest. **Methods**: This retrospective observational study included patients who experienced out-of-hospital cardiac arrest and were transported by emergency medical services between September 2021 and December 2022. We analyzed run sheets, the cardiac arrest registry, and Out-of-Hospital Cardiac Arrest Surveillance data, which contain patient demographics, time variables, Utstein factors, posthospital arrival treatments, and clinical outcomes. The primary outcome was emergency department mortality. The secondary outcome was poor neurological outcome (cerebral performance category scores 3–5). Logistic regression and mediation analyses assessed associations between protocol violations, transport times, and clinical outcomes. **Results**: Among the 3474 cardiac arrest cases, 1534 (44.2%) had transport protocol violations. Violations were associated with longer scene and transfer times. The emergency department survival rates for the protocol-violation and -nonviolation groups were 15.4% and 16.4%, respectively. Protocol violations were not associated with mortality (odds ratio [OR]: 1.04; 95% confidence interval [CI] 0.85–1.27, *p* = 0.70) or poor neurological outcomes (OR: 1.00; 95% CI 0.45–2.18, *p* = 0.99). Mediation analysis revealed that increased transfer time did not affect clinical outcomes. **Conclusions**: These results suggest that transport strategies should consider real-time availability of emergency resources and adopt an evidence-based approach.

## 1. Introduction

Among patients with out-of-hospital cardiac arrest (OHCA), the proportion of patients who achieve return of spontaneous circulation (ROSC) at the scene through advanced life support (ALS) ranges from 10% to 40%, depending on regional and clinical conditions [1,2,3,4,5]. Although patients who do not achieve ROSC have low survival rates, evidence suggests that appropriate transport can improve survival. Therefore, structured transport strategies should be established [1,4,6]. In emergency medical service (EMS) settings, providing ALS, which includes extracorporeal membrane oxygenation, at the scene is challenging; timely transport to a hospital with skilled professionals is critical [7,8].

The 2021 European Resuscitation Council guidelines emphasize the importance of systems with defined criteria for early transport to hospital in the management of patients with OHCA [9]. A prospective study using a large OHCA registry reported that prolonged on-scene treatment is associated with decreased 30-day survival rates [10]. Another study used a generalized additive model to determine a transfer time cutoff of 7.5 min for predicting favorable neurological outcomes [11]. The transport of patients experiencing intra-arrest must consider time-critical clinical circumstances. As such, determining the timing of transport initiation and selecting a hospital based on distance are major challenges [12,13].

Protocols depend on regional governance and available EMS resources, and clear scientific evidence on whether patients should be transported to the nearest hospital or a specialized cardiac arrest center is lacking [11,14,15,16]. Furthermore, few studies have investigated transport strategies based on ROSC status in the prehospital phase. Few studies have categorized prehospital duration based on specific events or investigated the time limits for each phase [17].

In a metropolitan area of the Republic of Korea, the government has implemented guidelines for selecting destination hospitals for patients with OHCA based on the closest distance in response to the difficulties in selecting hospitals following the coronavirus disease 2019 (COVID-19) pandemic. This protocol stipulates that patients with OHCA should be transported to the nearest emergency department (ED) that can handle cardiac arrest cases from the location of the incident. The EMS dispatch center receives updates every two hours regarding the capacity of each hospital to accept patients and incorporates this information into the process. Using this data, a rule-based system is used to select destination hospitals through a four-step process when the nearest ED is unavailable. This protocol assumes that selecting the closest hospital is practical and effective because of the high concentration of EDs capable of handling cardiac arrest in the city.

This study evaluated the impact of transport time delays caused by protocol violations on the clinical outcomes of patients who experienced intra-arrest in a metropolitan area where EMSs select hospitals using the shortest-distance protocol to minimize prehospital transport time.

## 2. Materials and Methods

### 2.1. Study Settings and Design

This observational study retrospectively analyzed data extracted from two nationwide datasets that were prospectively collected under the supervision of the National Fire Agency and the Korea Disease Control and Prevention Agency.

This study was approved by the Institutional Review Board of a Severance hospital in the Republic of Korea (Approval No. 4-2024-0637; approved 12 July 2024). The requirement for informed consent was waived because the patient data were anonymized. This study adhered to the ethical principles of the Declaration of Helsinki and followed the STROBE guidelines.

The Republic of Korea’s public EMS system is an ambulance service that provides basic life support and is exclusively operated by the National Fire Agency. Public EMS providers can perform cardiopulmonary resuscitation (CPR) using an automated external defibrillator onsite and during transport. Additionally, they can administer intermediate-level interventions such as endotracheal intubation and intravenous injections under direct medical guidance. However, public EMS providers do not have the authority to declare death officially, and all patients with OHCA are transported to the ED unless a medical director confirms death, following the national EMS CPR protocol. Seoul, the setting of our study, is the capital city of the Republic of Korea, with a population of 9.37 million and a metropolitan area covering 605 km^2^. In 2022, there were 4634 OHCA transport cases in Seoul, corresponding to 49.4 cases per 100,000 people. Seoul has 67 operational emergency medical institutions (level 1 EDs = 7, level 2 EDs = 24, level 3 EDs = 35). An EMS dispatch center supervises 24 regional fire stations that manage 161 ambulances and oversees all public EMS operations [18]. The city is administratively divided into four major regions based on geographical criteria, and EMSs are regionalized and operated around the available fire stations and hospitals in each region.

### 2.2. Implementation of the Four-Tiered Transport Protocol for Patients Experiencing Intra-Arrest

**Figure 1:**
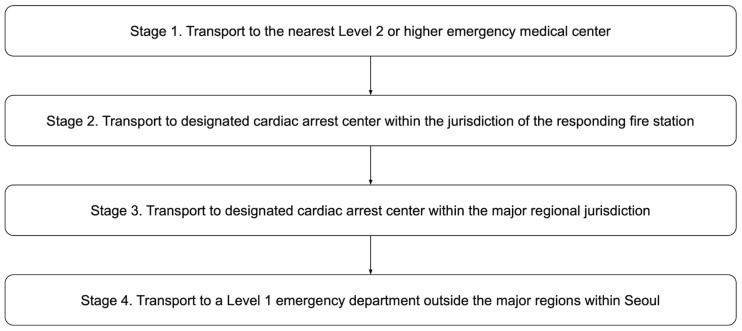
Four-tiered transport protocol for patients experiencing intra-arrest.

Seoul has implemented a four-tier transport protocol since September 2021 to ensure rapid and appropriate hospital selection. This protocol maximizes the EMS system efficiency and optimizes medical care for patients with OHCA before hospital arrival. Following the COVID-19 pandemic, ED resource saturation and shutdowns made it challenging to select hospitals capable of immediately treating patients with cardiac arrest. The city government developed this protocol to address these challenges. Each stage of the protocol is presented in Figure 1. All EDs in Seoul must notify the EMS dispatch center and request ambulance diversion if they cannot provide resuscitation for patients with cardiac arrest because of medical personnel, equipment, or facility shortages. This notification remains valid for 2 h and is automatically lifted unless an additional request is made. If all EDs corresponding to a given protocol stage are unavailable, the protocol advances to the next stage. When all EDs across Seoul cannot accommodate a patient, continuous CPR is maintained in the ambulance while awaiting further instructions and alternative arrangements.

### 2.3. Study Population

We included adult patients aged 18 years or older with OHCA who were consecutively reported to the public EMS in Seoul between September 2021 and December 2022. Patients whose cardiac arrest was witnessed by EMS personnel, transported by helicopter, or whose cardiac arrest was due to traumatic causes were excluded from the study. Additionally, cases in which information about arrest location or clinical outcomes could not be verified were excluded.

### 2.4. Data Source and Variables

The data were extracted from the following sources: EMS run sheets and the EMS cardiac arrest registry managed by the National Fire Agency and Out-of-Hospital Cardiac Arrest Surveillance data managed by the Korea Disease Control and Prevention Agency. The EMS run sheets include patient demographic information, time variables for all prehospital phases from emergency call activation to hospital arrival, and information on the receiving hospital. The EMS cardiac arrest registry contains information about the time of patient collapse, Utstein factors, administration of CPR and defibrillation by EMS providers, and out-of-hospital treatment outcomes. The Out-of-Hospital Cardiac Arrest Surveillance data include information on posthospital arrival treatments such as percutaneous coronary intervention or targeted temperature management, survival status at ED discharge, and neurological outcomes. These three data sources were merged to create the final study dataset.

### 2.5. Variable Definitions and Measurements

The primary exposure, protocol violation, was defined as a failure to transport the patient to the nearest ED designated by the protocol that was operationally available and not on ambulance diversion status when EMS departed the scene. The “direction feature” of the Naver Map application (Naver Corp., Seongnam, Republic of Korea; https://map.naver.com, accessed on 30 January 2026) was used to search for the routes from the cardiac arrest location to EDs. The nearest ED was determined based on the shortest route rather than the straight-line distance. The vehicle type was set to a mid-sized van. The prehospital time was divided into four phases according to the times recorded in the EMS run sheet: reaction time (from cardiac arrest to EMS call), dispatch time (from call to arrival at the scene), scene time (from arrival at the scene to departure from the scene), and transfer time (from departure from the scene to arrival at the receiving hospital). Off time was defined as weekdays from 6:00 p.m. to 9:00 a.m. the following day, as well as weekends and public holidays. Multiple cardiac arrests were defined as two or more cardiac arrests occurring within 60 min in the same ambulance catchment area. High-capacity hospitals were defined as those in the top 25% based on the number of patients who experienced intra-arrest admitted during the study period.

### 2.6. Outcome Measures

The primary outcome was ED mortality; the secondary outcome was poor neurological outcome. ED mortality was defined as a patient declared dead after arriving at the ED. A poor neurological outcome was defined as a cerebral performance category score of 3, 4 or 5. The outcomes were assessed by trained medical record reviewers from the Korea Disease Control and Prevention Agency and recorded in the registry.

### 2.7. Statistical Analysis

Categorical variables are reported as frequencies and percentages, whereas continuous variables are presented as the means ± standard deviations. Differences in continuous variables between the two groups were analyzed using Student’s *t* test, and differences in categorical variables were analyzed using the chi-squared test. A *p* value < 0.05 was considered significant. A univariable analysis was conducted to compare the baseline characteristics of the groups based on protocol violation status. A multivariable logistic regression analysis was performed with *p* values < 0.05 in the univariable analysis. The significance of the primary and secondary outcomes according to protocol violation status was assessed using a multivariable logistic regression analysis. As a post hoc, hypothesis-driven extension of the primary analysis, we conducted a mediation analysis to granularly examine whether the violation-associated increase in transfer time could explain the absence of an association between protocol violation and clinical outcomes [19]. This analysis aimed to decompose the overall null effect observed in the logistic models by assessing whether the specific time delay attributable to protocol violation exerted any indirect influence on ED mortality or neurological outcomes. The indirect effect through transfer time—the only prehospital interval significantly affected by violation—was estimated and summarized using the coefficient and its standard error. All statistical analyses were performed using SAS version 9.4 (SAS Institute Inc., Cary, NC, USA). Mediation analyses were conducted using R version 4.5.1 (The R Foundation for Statistical Computing, Vienna, Austria) with the lavaan package 0.6-19 (Ghent University, Ghent, Belgium).

## 3. Results

**Figure 2:**
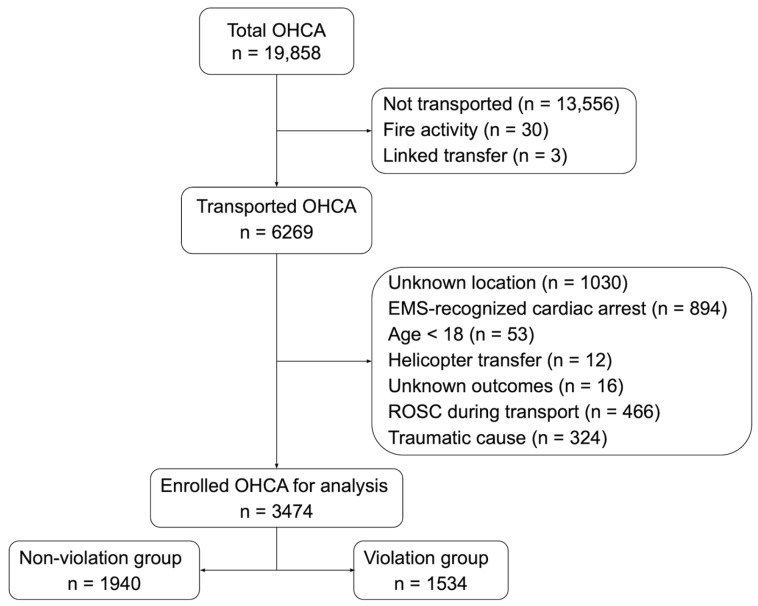
Flow diagram of patient selection. EMS, emergency medical service; OHCA, out-of-hospital cardiac arrest; ROSC, return of spontaneous circulation.

Of the 19,858 cardiac arrest-related calls made during the study period, 6269 patients were transported to the hospital by EMS. After applying the exclusion criteria, 3474 patients with OHCA were analyzed (Figure 2).

Table 1 presents the demographics of all patients, as well as the protocol nonviolation and violation groups. Among the 3474 cases, 1534 cases (44.2%) violated the transport protocol. The emergency department survival rates in the protocol-violation and -nonviolation groups were 15.4% and 16.4%, respectively. The total time from cardiac arrest to hospital arrival did not differ significantly between the nonviolation and violation groups. The violation group showed significantly longer scene and transfer times. Among the four major regions, the southwestern and northwestern areas had higher protocol violation rates, at 49.8% and 49.1%, respectively. Among the 42 hospitals, 11 were high-capacity hospitals; 57.4% of all patients were transferred to these hospitals, of which 53.7% were in the nonviolation group and 62.2% were in the violation group.

**Figure 3:**
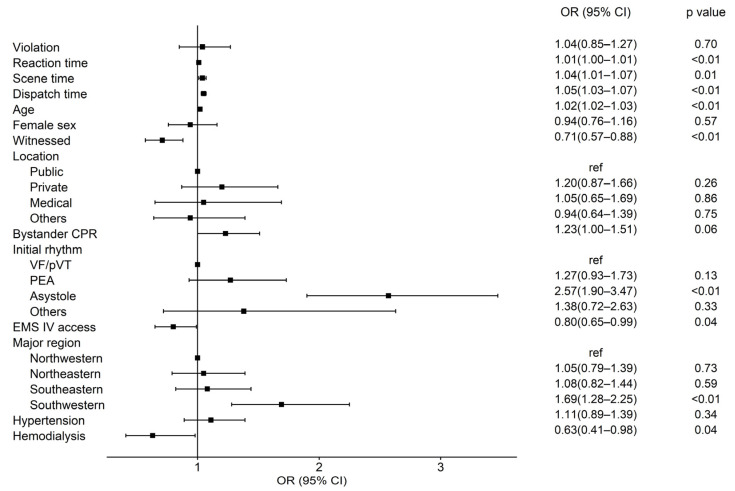
Forest plot of multivariable logistic regression analysis for factors associated with ED mortality. OR, odds ratio; CI, confidence interval; CPR, cardiopulmonary resuscitation; VF, ventricular fibrillation; pVT, pulseless ventricular tachycardia; PEA, pulseless electrical activity; EMS, emergency medical service; IV, intravenous.

Figure 3 and Supplementary Table S1 present the results of the multivariable logistic regression analysis for factors associated with ED mortality among patients who did not achieve prehospital ROSC. Several established prognostic factors remained independently associated with increased ED mortality, including older age (adjusted odds ratio [aOR]: 1.02; 95% CI 1.02–1.03, *p* < 0.01), non-shockable initial rhythms (Asystole: aOR: 2.57; 95% CI 1.90–3.47, *p* < 0.01), and unwitnessed arrest (aOR: 0.71 for witnessed; 95% CI 0.57–0.88, *p* < 0.01). Protocol violation was not significantly associated with ED mortality (aOR: 1.04; 95% CI 0.85–1.27, *p* = 0.70).

**Figure 4:**
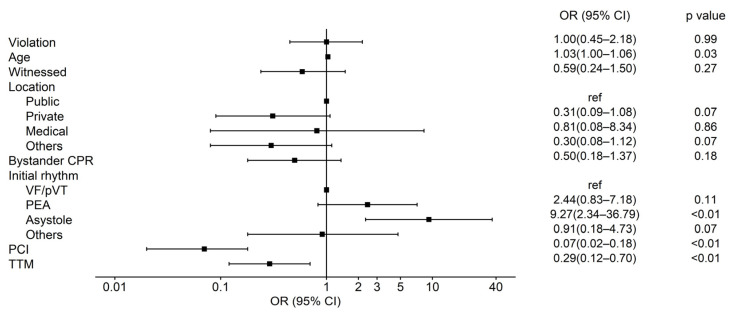
Forest plot of multivariable logistic regression analysis for factors associated with poor neurologic outcomes. OR, odds ratio; CI, confidence interval; CPR, cardiopulmonary resuscitation; VF, ventricular fibrillation; pVT, pulseless ventricular tachycardia; PEA, pulseless electrical activity; PCI, percutaneous coronary intervention; TTM, targeted temperature management.

The multivariable analysis for poor neurological outcomes, as shown in Figure 4 and Supplementary Table S2, also demonstrated consistency with established prognosticators. Older age (aOR: 1.03; 95% CI 1.00–1.06, *p* = 0.03), non-shockable initial rhythm (Asystole: aOR: 9.27; 95% CI 2.34–36.79, *p* < 0.01), and the post-resuscitation interventions—such as PCI (aOR: 0.07; 95% CI 0.02–0.18, *p* < 0.01) and TTM (aOR: 0.29; 95% CI 0.12–0.70, *p* < 0.01)—were independently associated. In this context, protocol violation showed no significant association with poor neurological prognosis (aOR: 1.00; 95% CI 0.45–2.18, *p* = 0.99).

**Figure 5:**
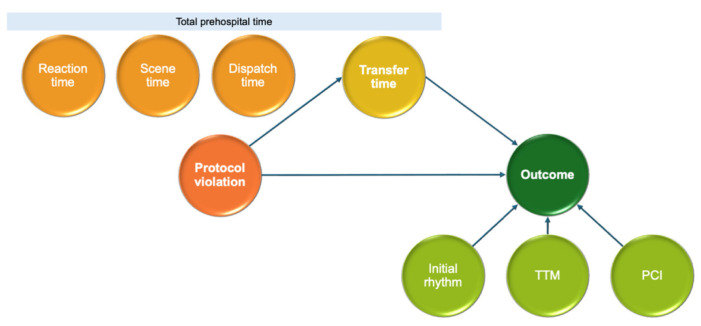
Path diagram of the mediation analysis.

Figure 5 and Table 2 present the mediation analysis results for the indirect effects of transport protocol violations on ED mortality and neurological outcomes. Protocol violations were significantly associated with prolonged transfer times (comparable coefficient = 0.93, *p* < 0.01). However, mediation analysis showed that the indirect effects of protocol violation on ED mortality and poor neurological outcome through transfer time were not statistically significant. These findings indicate that although protocol violations resulted in longer transport durations, this specific delay did not translate into worse clinical outcomes in this metropolitan setting.

A sensitivity analysis was conducted on a prognostically favorable subgroup (n = 319; patients with shockable initial rhythms) to mitigate potential bias arising from the high baseline mortality of the overall cohort. Although protocol violations significantly increased transfer times in this subgroup (standardized coefficients of 0.79 and 0.76, respectively; both *p* < 0.01), mediation analysis showed no significant indirect influence on clinical outcomes. Specifically, the indirect effect was 0.01 for ED mortality with a *p*-value of 0.85 and −0.06 for poor neurologic outcome with a *p*-value of 0.68, as detailed in Supplementary Figure S1.

## 4. Discussion

The present study, which was conducted in a metropolitan area in the Republic of Korea, revealed that while a transport protocol for selecting the closest hospital to minimize transport times was frequently violated, the delays in transport times caused by these violations did not affect clinical outcomes. The prehospital time for patients with OHCA can be categorized into four sequential events: reaction time, dispatch time, scene time, and transfer time. As the current protocol is intended to minimize transfer time from departure at the scene to hospital arrival, protocol violations are associated only with increased transfer time rather than total prehospital time. In our study, total prehospital time did not differ between the protocol-violation and -nonviolation groups, despite the violation group having a significantly longer transfer time. The main reason protocol violations did not significantly affect the clinical outcomes of patients who experienced intra-arrest is likely because the total time from arrest to hospital arrival remained unchanged. This observation suggests that the delays incurred during the transfer phase might have been offset by other temporal variables, although we cannot exclude the influence of unmeasured factors such as the time taken for destination decision-making at the scene.

Subsequently, we performed a path analysis to assess the direct and indirect effects of longer transfer times on clinical outcomes. These findings suggest that in this metropolitan setting, a transport strategy strictly prioritized on the shortest distance may not necessarily translate into improved ED survival or neurological outcomes for patients experiencing intra-arrest. While this interpretation warrants caution—as outcomes could be influenced by unmeasured confounders, including variations in the quality of post-cardiac arrest care—it nonetheless provides important insights into the practical effectiveness of current distance-based protocols. A review of hospital selection strategies for patients with OHCA reveals that the outcomes vary depending on community conditions and governance. Seoul covers 605.2 km^2^ and has 49 hospitals that can admit patients experiencing intra-arrest, a relatively high density of cardiac arrest centers. In areas with a high density of cardiac arrest centers, not choosing the closest hospital is unlikely to delay transfer times. Cournoyer et al. [20] reported that if total prehospital time is delayed by more than 14 min, the potential benefits of transport to a cardiac arrest center are offset. In our study, the transfer time in the violation group was 3 min longer than that in the nonviolation group, which was unlikely to be long enough to affect the survival or neurological outcomes of the patients who experienced intra-arrest. Similarly, the ARREST trial, which was also conducted in a metropolitan area with a well-developed cardiac arrest network and resource-rich hospitals, failed to demonstrate the clinical benefits of bypass transport to cardiac arrest centers [21]. However, studies addressing the same issue in regions with different geographic and healthcare characteristics have reported contrasting results [22,23]. Therefore, in regions where hospitals capable of admitting patients experiencing intra-arrest are sparsely distributed, leading to inherently longer transfer times, the findings may differ.

Our study focused exclusively on patients who experienced intra-arrest, excluding those who achieved ROSC in the prehospital phase or whose cardiac arrest was caused by trauma. Patients who do not achieve ROSC in the prehospital phase may experience more severe post-resuscitation syndrome due to prolonged ischemia, necessitating a different approach to hospital selection than that for those who have regained spontaneous circulation [17]. However, existing studies have not provided sufficient evidence to establish transport protocols exclusively for patients experiencing intra-cardiac arrest [17]. Therefore, our results offer insights into transport strategies for patients who fail to achieve ROSC in the field, particularly in regions with a high concentration of cardiac arrest care resources.

Our study revealed that significantly more violation cases occurred in high-capacity hospitals. This finding suggests that prehospital providers prefer to transport patients to hospitals with higher acceptance rates despite violating established protocols. Prehospital providers lack specialized expertise and face legal restrictions on their clinical autonomy, while decisions regarding hospital selection for patients experiencing intra-arrest are constrained by time and physical limitations. Therefore, an optimized protocol is necessary to guide their decision-making [14]. The hospital selection protocol in this study was strictly rule-based, primarily prioritizing the closest facility. However, this approach inherently overlooks the clinical heterogeneity of patients and the non-cooperative, dynamic circumstances on-scene that pre-hospital providers must navigate. Furthermore, the reliance on 2-hourly updates for hospital availability introduces a degree of temporal uncertainty, as the reported capacity may not align with the real-time status during an emergency. Our findings—showing a preference for high-capacity hospitals despite protocol violations—underscore the need for more flexible, real-time information-sharing strategies that empower providers to make optimized decisions in a complex operational environment [24,25]. Incorporating community-specific characteristics into the protocol, when delays from non-shortest-distance transfers may not adversely affect patient outcomes, and leveraging information and communication technologies to verify hospital acceptance in real time could support the development of a more practical transport strategy.

Our study has several limitations. First, given the retrospective nature of our study using data collected after protocol implementation, our findings may be subject to inherent biases, including the absence of early biological markers of patient vulnerability and the failure to fully capture operational complexities—such as on-scene confounders and hospital status updates—which may have influenced both protocol adherence and clinical outcomes [26]. Also, the high baseline mortality (84.0%) and poor neurological outcome rate (98.9%) in our intra-arrest cohort likely introduced a ceiling effect, limiting the statistical power to detect subtle differences between groups. Consequently, these findings apply specifically to a high-acuity subgroup failing to achieve prehospital ROSC. Second, in regions with a low density of hospitals equipped to receive patients with cardiac arrest, variations in transfer times may affect time to ROSC and the quality of post-resuscitation care, potentially leading to different outcomes than those observed in our study. Moreover, our results should be interpreted as an observed lack of independent association within a high-density urban system, rather than definitive proof of no effect. Therefore, these findings should be interpreted within the context of settings that offer high resource availability for cardiac arrest management. Third, although prehospital Advanced Cardiovascular Life Support interventions, high-quality chest compressions, and time to reperfusion are known to influence clinical outcomes in cardiac arrest patients, in our study region, these interventions were authorized only for select public EMS providers. Furthermore, ED mortality and neurological status at discharge are proximal endpoints that may not reflect long-term functional recovery, although they are most directly influenced by prehospital transport strategies. Consequently, our results apply specifically to a high-acuity subgroup failing to achieve prehospital ROSC. Finally, data on the quality of chest compressions and time to reperfusion were unavailable; therefore, caution is warranted when interpreting our results.

## 5. Conclusions

In summary, our study results revealed that a rule-based protocol for transporting patients experiencing intra-arrest to the closest cardiac arrest center in a metropolitan setting was frequently violated. However, these violations did not lead to worse clinical outcomes. Our findings suggest that transport strategies for patients experiencing intra-arrest should consider the availability of emergency resources in the communities and adopt an evidence-based approach to improve clinical outcomes.

## Figures and Tables

**Table 1:** Participant demographics.

		Total n (%)	Nonviolation Group n (%)	Violation Group n (%)	*p* Value
Total patients		3474	1940 (55.8)	1534 (44.2)	
Age		70.9 ± 14.6	71.1 ± 14.4	70.6 ± 14.8	0.27
Sex	Males	2211 (63.6)	1196 (61.6)	1015 (66.2)	<0.01
Witnessed	Yes	1643 (48.7)	898 (47.7)	745 (49.9)	0.20
Location					0.89
	Public	294 (8.5)	158 (8.1)	136 (8.9)	
	Private	2602 (74.9)	1456 (75.1)	1146 (74.7)	
	Medical	231 (6.7)	130 (6.7)	101 (6.6)	
	Others	347 (10.0)	196 (10.1)	151 (9.8)	
Bystander CPR	Yes	2129 (61.9)	1182 (61.6)	947 (62.2)	0.71
Initial rhythm					0.14
	VF/pVT	319 (9.2)	168 (8.7)	151 (9.8)	
	PEA	844 (24.3)	497 (25.6)	347 (22.6)	
	Asystole	2240 (64.5)	1239 (63.9)	1001 (65.3)	
	Others	71 (2.0)	36 (1.9)	35 (2.3)	
EMS advanced airway	Yes	3371 (97.0)	1883 (97.1)	1488 (97.0)	0.92
EMS IV access	Yes	2175 (62.6)	1169 (60.3)	1006 (65.6)	<0.01
EMS epinephrine	Yes	474 (13.7)	264 (13.6)	210 (13.7)	0.95
PCI	Yes	146 (4.2)	85 (4.4)	61 (4.0)	0.55
TTM	Yes	233 (6.7)	142 (7.3)	91 (5.9)	0.10
Total time	min	61.4 ± 104.5	61.0 ± 106.3	61.8 ± 102.3	0.81
Reaction time	min	30.3 ± 104.3	31.8 ± 106.0	28.4 ± 102.2	0.34
Scene time	min	8.7 ± 3.8	8.7 ± 3.7	8.9 ± 3.9	0.11
Dispatch time	min	15.3 ± 5.0	14.9 ± 4.8	15.8 ± 5.2	<0.01
Transfer time	min	7.7 ± 4.0	6.3 ± 3.1	9.4 ± 4.3	<0.01
ED mortality		2919 (84.0)	1622 (83.6)	1297 (84.6)	0.45
Hospital mortality		391 (78.2)	231 (79.7)	160 (76.2)	0.35
Poor neurological outcome		3436 (98.9)	1919 (98.9)	1517 (98.9)	0.94
Major region					<0.01
	Northeastern	876 (25.2)	517 (26.7)	359 (23.4)	
	Southeastern	846 (24.4)	538 (27.7)	308 (20.1)	
	Southwestern	1049 (30.2)	527 (27.2)	522 (34.0)	
	Northwestern	703 (20.2)	358 (18.5)	345 (22.5)	
Off time transfer	Yes	2214 (63.9)	1239 (64.0)	975 (63.6)	0.81
Multiple cardiac arrests	Yes	61 (1.8)	37 (1.9)	24 (1.6)	0.45
High-capacity hospitals	Yes	1995 (57.4)	1041 (53.7)	954 (62.2)	<0.01
Hypertension	Yes	1254 (36.1)	695 (35.8)	559 (36.4)	0.71
Diabetes	Yes	986 (28.4)	557 (28.7)	429 (28.0)	0.63
Cerebrovascular disease	Yes	327 (9.4)	189 (9.7)	138 (9.0)	0.45
Pulmonary disease	Yes	288 (8.3)	140 (7.2)	148 (9.7)	<0.01
Cardiac disease	Yes	552 (15.9)	294 (15.2)	258 (16.8)	0.18
Tuberculosis	Yes	13 (0.4)	8 (0.4)	5 (0.3)	0.68
Hepatitis	Yes	4 (0.1)	1 (0.1)	3 (0.2)	0.33
Liver cirrhosis	Yes	54 (1.6)	27 (1.4)	27 (1.8)	0.38
Allergy	Yes	3 (0.1)	2 (0.1)	1 (0.1)	1.00
Malignancy	Yes	445 (12.8)	268 (13.8)	177 (11.5)	0.046
Chronic kidney disease	Yes	222 (6.4)	116 (6.0)	106 (6.9)	0.27
Hemodialysis	Yes	138 (4.0)	77 (4.0)	61 (4.0)	0.99

CPR, cardiopulmonary resuscitation; ED, emergency department; EMS, emergency medical service; IV, intravenous; PCI, percutaneous coronary intervention; PEA, pulseless electrical activity; pVT, pulseless ventricular tachycardia; TTM, targeted temperature management; VF, ventricular fibrillation.

**Table 2:** Mediation analysis.

Outcomes	ED Mortality			Poor Neurological Outcome		
Mediation Effect	Estimate	95% CI	*p* Value	Estimate	95% CI	*p* Value
Model with mediator (Protocol violation)						
Total effect	0.08	−0.09–0.25	0.34	0.28	−0.30–0.86	0.35
Direct effect	−0.03	−0.16–0.10	0.64	0.17	−0.35–0.69	0.52
Indirect effect	0.05	−0.01–0.12	0.11	−0.11	−0.26–0.04	0.16

ED, emergency department; CI, confidence interval.

## Data Availability

The datasets used in this study were obtained from the National Fire Agency and the Korea Disease Control and Prevention Agency. Owing to data use agreements and institutional policies, these datasets are not publicly available and cannot be shared by the authors.

## Supplementary Materials

The following supporting information can be downloaded at https://www.mdpi.com/article/10.3390/jcm15031282/s1, Table S1: Associations between violations and ED mortality according to the logistic regression analyses; Table S2: Association between violation and poor neurological outcome in the logistic regression analyses; Figure S1: Mediation analysis for patient group with prehospital shockable rhythm.

## Abbreviations

The following abbreviations are used in this manuscript:

OHCA	Out-of-hospital cardiac arrest
ROSC	Return of spontaneous circulation
ALS	Advanced life support
EMS	Emergency medical service
ED	Emergency department
COVID-19	Coronavirus disease 2019
STROBE	Strengthening the Reporting of Observational Studies in Epidemiology
CPR	Cardiopulmonary resuscitation
VF	Ventricular fibrillation
pVT	Pulseless ventricular tachycardia
PEA	Pulseless electrical activity
IV	Intravenous
PCI	Percutaneous coronary intervention
TTM	Targeted temperature management
OR	Odds ratio
CI	Confidence interval
