# Supplementary material for: Impact of Violations of the Shortest Distance-Based Transport Protocol for Intra-Arrest on Clinical Outcomes in a Metropolitan City: A Large-Scale Registry Study
Source: J Clin Med. 2026 Feb 5;15(3):1282. doi: 10.3390/jcm15031282 (PMC12898337; doi:10.3390/jcm15031282)
Supplement: Supplementary file 1 [file jcm-15-01282-s001.zip › jcm-4099987-supplementary.pdf]

Journal of
Clinical Medicine

MDPI

**Table S1.** Associations between violations and ED mortality according to the logistic regression analyses.

| | Univariable | | | Multivariable | | |
|---|---|---|---|---|---|---|
| | OR | 95% CI | P value | OR | 95% CI | p value |
| Violation | 1.07 | 0.89–1.29 | 0.45 | 1.04 | 0.85–1.27 | 0.70 |
| Reaction time | 1.01 | 1.01–1.01 | <0.01 | 1.01 | 1.00–1.01 | <0.01 |
| Scene time | 1.03 | 1.01–1.06 | 0.02 | 1.04 | 1.01–1.07 | 0.01 |
| Dispatch time | 1.04 | 1.02–1.06 | <0.01 | 1.05 | 1.03–1.07 | <0.01 |
| Age | 1.03 | 1.02–1.03 | <0.01 | 1.02 | 1.02–1.03 | <0.01 |
| Female sex | 1.22 | 1.00–1.48 | 0.046 | 0.94 | 0.76-1.16 | 0.57 |
| Witnessed | 0.57 | 0.47–0.69 | <0.01 | 0.71 | 0.57–0.88 | <0.01 |
| Location | | | | | | |
| Public | ref | | | ref | | |
| Private | 1.95 | 1.46–2.60 | <0.01 | 1.20 | 0.87–1.66 | 0.26 |
| Medical | 1.49 | 0.97–2.30 | 0.07 | 1.05 | 0.65–1.69 | 0.86 |
| Others | 0.96 | 0.67–1.39 | 0.84 | 0.94 | 0.64–1.39 | 0.75 |
| Bystander CPR | 1.02 | 0.85–1.23 | 0.81 | 1.23 | 1.00–1.51 | 0.06 |
| Initial rhythm | | | | | | |
| VF/pVT | ref | | | ref | | |
| PEA | 1.65 | 1.25–2.20 | <0.01 | 1.27 | 0.93–1.73 | 0.13 |
| Asystole | 3.96 | 3.02–5.18 | <0.01 | 2.57 | 1.90–3.47 | <0.01 |
| Others | 1.66 | 0.91–3.04 | 0.10 | 1.38 | 0.72–2.63 | 0.33 |
| EMS advanced airway | 1.20 | 0.72–1.99 | 0.49 | | | |
| EMS IV access | 0.83 | 0.68–1.00 | 0.049 | 0.80 | 0.65–0.99 | 0.04 |
| EMS epinephrine | 1.03 | 0.79–1.35 | 0.82 | | | |
| Major region | | | | | | |
| Northwestern | ref | | | ref | | |
| Northeastern | 1.14 | 0.88–1.48 | 0.32 | 1.05 | 0.79–1.39 | 0.73 |
| Southeastern | 1.06 | 0.82–1.38 | 0.67 | 1.08 | 0.82–1.44 | 0.59 |
| Southwestern | 1.48 | 1.14–1.93 | <0.01 | 1.69 | 1.28–2.25 | <0.01 |
| Off time transfer | 0.97 | 0.80–1.17 | 0.73 | | | |
| Multiple cardiac arrest | 1.26 | 0.60–2.67 | 0.54 | | | |
| High-capacity hospitals | 0.99 | 0.82–1.19 | 0.90 | | | |
| Hypertension | 1.42 | 1.17–1.73 | <0.01 | 1.11 | 0.89-1.39 | 0.34 |
| Diabetes | 1.03 | 0.84–1.26 | 0.80 | | | |
| Cerebrovascular disease | 1.18 | 0.85–1.63 | 0.32 | | | |
| Pulmonary disease | 1.06 | 0.76–1.48 | 0.74 | | | |
| Cardiac disease | 1.09 | 0.85–1.40 | 0.51 | | | |

| | | | | | | |
|---|---|---|---|---|---|---|
| Tuberculosis | 2.29 | 0.30–17.62 | 0.43 | | | |
| Hepatitis | 1.71 | 0.07–44.51 | 0.75 | | | |
| Liver cirrhosis | 0.95 | 0.46–1.95 | 0.89 | | | |
| Allergy | 0.38 | 0.03–4.20 | 0.43 | | | |
| Malignancy | 1.43 | 1.07–1.92 | 0.02 | | | |
| Chronic kidney disease | 0.75 | 0.54–1.06 | 0.11 | | | |
| Hemodialysis | 0.57 | 0.38–0.84 | <0.01 | 0.63 | 0.41–0.98 | 0.04 |

CI, confidence interval; CPR, cardiopulmonary resuscitation; ED, emergency department; EMS, emergency medical service; IV, intravenous; OR, odds ratio; PEA, pulse-less electrical activity; pVT, pulseless ventricular tachycardia; VF, ventricular fibrillation.

**Table S2.** Association between violation and poor neurological outcome in the logistic regression analyses.

| | Univariable | | | Multivariable | | |
|---|---|---|---|---|---|---|
| | OR | 95% CI | P value | OR | 95% CI | p value |
| Violation | 0.98 | 0.51–1.86 | 0.94 | 1.00 | 0.45–2.18 | 0.99 |
| Reaction time | 1.04 | 1.00–1.08 | 0.07 | | | |
| Scene time | 1.08 | 0.97–1.20 | 0.18 | | | |
| Dispatch time | 1.07 | 0.99–1.15 | 0.08 | | | |
| Age | 1.05 | 1.03–1.07 | <0.01 | 1.03 | 1.00–1.06 | 0.03 |
| Female sex | 1.61 | 0.78–3.32 | 0.20 | | | |
| Witnessed | 0.28 | 0.13–0.61 | <0.01 | 0.59 | 0.24–1.50 | 0.27 |
| Location | | | | | | |
| Public | ref | | | ref | | |
| Private | 1.70 | 0.58–4.97 | 0.34 | 0.31 | 0.09–1.08 | 0.07 |
| Medical | 3.17 | 0.35–28.58 | 0.30 | 0.81 | 0.08–8.34 | 0.86 |
| Others | 0.39 | 0.12–1.21 | 0.10 | 0.30 | 0.08–1.12 | 0.07 |
| Bystander CPR | 0.36 | 0.16–0.83 | 0.02 | 0.50 | 0.18–1.37 | 0.18 |
| Initial rhythm | | | | | | |
| VF/pVT | ref | | | ref | | |
| PEA | 10.17 | 4.35–23.76 | <0.01 | 2.44 | 0.83–7.718 | 0.11 |
| Asystole | 63.40 | 19.03–211.25 | <0.01 | 9.27 | 2.34–36.79 | <0.01 |
| Others | 1.93 | 0.57–6.57 | 0.29 | 0.91 | 0.18–4.73 | 0.07 |
| EMS advanced airway | 0.88 | 0.12–6.50 | 0.90 | | | |
| EMS IV access | 0.98 | 0.50–1.90 | 0.94 | | | |
| EMS epinephrine | 0.70 | 0.31–1.59 | 0.39 | | | |
| PCI | 0.02 | 0.01–0.03 | <0.01 | 0.07 | 0.02–0.18 | <0.01 |
| TTM | 0.05 | 0.03–0.10 | <0.01 | 0.29 | 0.12–0.70 | <0.01 |
| Major region | | | | | | |
| Northwestern | ref | | | | | |
| Northeastern | 2.19 | 0.64–7.52 | 0.21 | | | |
| Southeastern | 0.52 | 0.21–1.28 | 0.15 | | | |

| | | | |
|---|---|---|---|
| Southwestern | 0.95 | 0.37–2.46 | 0.91 |
| Off time transfer | 1.03 | 0.53–2.00 | 0.93 |
| Multiple cardiac arrest | 1.40 | 0.08–23.61 | 0.81 |
| High-capacity hospitals | 1.35 | 0.71–2.57 | 0.35 |
| Hypertension | 1.23 | 0.62–2.44 | 0.56 |
| Diabetes | 1.29 | 0.60–2.71 | 0.52 |
| Cerebrovascular disease | 1.88 | 0.45–7.85 | 0.39 |
| Pulmonary disease | 0.77 | 0.27–2.17 | 0.62 |
| Cardiac disease | 0.84 | 0.37–1.91 | 0.67 |
| Tuberculosis | 0.30 | 0.02–5.78 | 0.43 |
| Hepatitis | 0.10 | 0.00–2.70 | 0.17 |
| Liver cirrhosis | 1.24 | 0.07–20.99 | 0.88 |
| Allergy | 0.08 | 0.00–2.44 | 0.15 |
| Malignancy | 11.47 | 0.70–187.54 | 0.09 |
| Chronic kidney disease | 2.54 | 0.35–18.60 | 0.36 |
| Hemodialysis | 3.25 | 0.20–53.71 | 0.41 |

CI, confidence interval; CPR, cardiopulmonary resuscitation; ED, emergency department; EMS, emergency medical service; IV, intravenous; OR, odds ratio; PCI, percutaneous coronary intervention; PEA, pulseless electrical activity; pVT, pulseless ventricular tachycardia; TTM, targeted temperature management; VF, ventricular fibrillation.

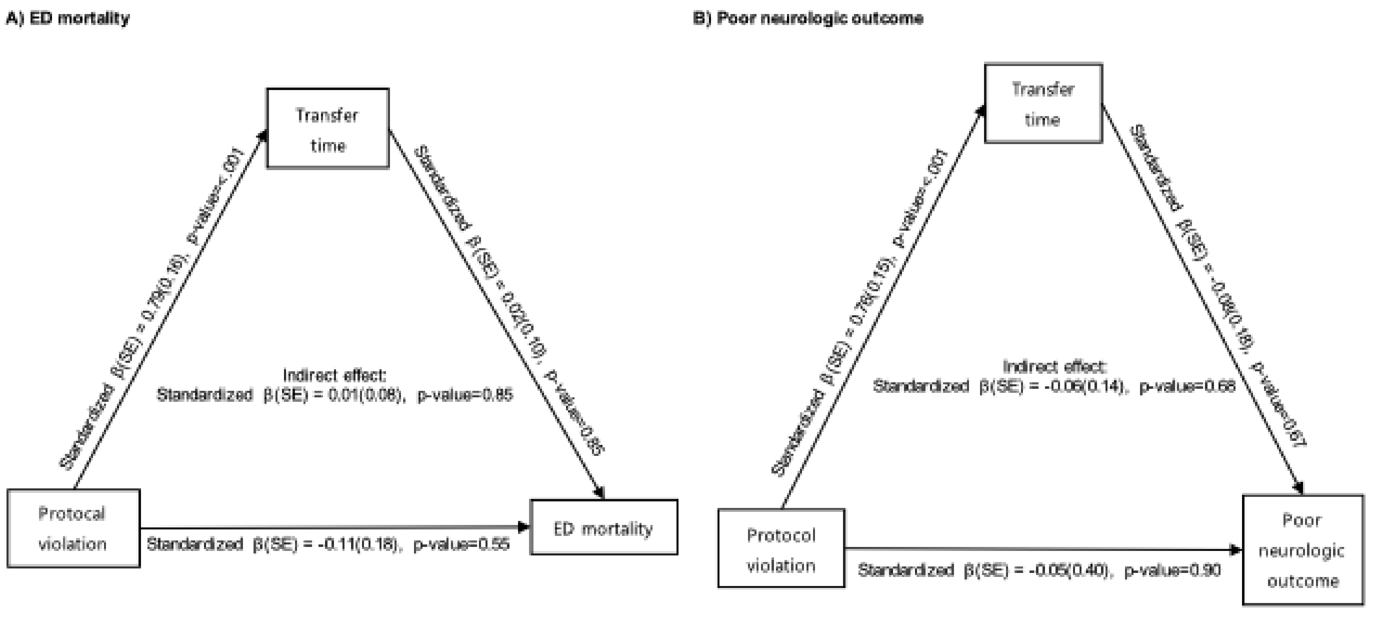


| Effect | Estimate | SE | 95%CI lower | 95%CI upper | p-value | Effect | Estimate | SE | 95%CI lower | 95%CI upper | p-value |
|---|---|---|---|---|---|---|---|---|---|---|---|
| Total effect | 0.12 | 0.22 | -0.31 | 0.56 | 0.58 | Total effect | 0.11 | 0.37 | -0.62 | 0.84 | 0.78 |
| Mediation effect | 0.01 | 0.08 | -0.14 | 0.17 | 0.85 | Mediation effect | -0.06 | 0.14 | -0.32 | 0.21 | 0.68 |
| Direct effect | -0.11 | 0.18 | -0.46 | 0.25 | 0.55 | Direct effect | -0.05 | 0.4 | -0.82 | 0.73 | 0.9 |
| Proportion mediated | 0.12 | 0.51 | -0.88 | 1.12 | 0.82 | Proportion mediated | 0.54 | 2.28 | -3.93 | 5.03 | 0.81 |

**Figure S1**. Mediation analysis for patient group with prehospital shockable rhythm
ED, emergency department.
